# A Case of Successful Allogeneic Hematopoietic Stem Cell Transplantation in a Severely Underweight Patient with Aplastic Anemia

**DOI:** 10.1155/2024/2044820

**Published:** 2024-03-04

**Authors:** Lilija Banceviča, Andrius Žučenka

**Affiliations:** ^1^Faculty of Medicine, Rīga Stradiņš University, Riga, Latvia; ^2^Hematology, Oncology, and Transfusion Medicine Centre, Vilnius University Hospital Santaros Klinikos, Santariskiu Str. 2, LT-08661 Vilnius, Lithuania

## Abstract

Allogeneic hematopoietic stem cell transplantation (alloHSTC) is considered definitive and the most effective treatment for young patients diagnosed with severe aplastic anemia. Low body mass index (BMI) is known to be associated with poorer outcomes in stem cell transplantation and higher mortality risks. Malnutrition negatively affects the patient's ability to mobilize stem cells, therefore reducing patients' stem cell production, although the patient's nutritional status improvement with enteral and parenteral nutrition may reduce the risks of stem cell graft failure and graft-vs-host disease (GVHD) occurrence. The present report demonstrates a severely underweight patient with aplastic anemia and a BMI of 11 kg/m^2^ who was unsuccessfully treated with immunosuppressive therapy followed by alloHSTC.

## 1. Introduction

Aplastic anemia is a nonmalignant commonly severe hematological disease, which is generally caused by autoimmune mechanisms, in some cases due to genetical mutations, such as inherited bone marrow failure syndromes (Fanconi anemia, Schwachman-Diamond syndrome, dyskeratosis congenita, and Diamond-Blackfan anemia). Elderly patients are treated with immunosuppressive therapy, including antithymocyte globulin (ATG), cyclosporine A (CsA), combined therapy of ATG + CsA + eltrombopag, and steroids (prednisone, dexamethasone), in patients younger than 40 years old. alloHSCT is a first-line treatment in cases of treating severe aplastic anemia. A low BMI index may negatively affect hematopoietic stem cell production after receiving an alloHSCT due to differentiation-associated pancytopenia, common in severely underweight patients without bone marrow failure [1]. Current data on alloHSCT in patients with a BMI below 18 is variable and insufficient; therefore, further investigation is necessary to understand the outcomes of severely malnourished cachectic patients receiving high-dosage conditioning therapy followed by alloHSCT.

## 2. Case Presentation

A 22-year-old female presented to the Hematology Stem Cell Transplant ward at the Latvian Center of Oncology, Riga, Latvia, for persistent infections, epistaxis, and ecchymosis in June 2021. The patient had no dizziness symptoms; however, mild fatigue was present. On clinical examination, vital signs were stable. Initial laboratory results showed remarkable neutropenia (0.46 *∗* 10 ^ 9/L) and anemia (58 g/L), and the Plt count was in reference ranges (180 *∗* 10 ^ 9/L). A peripheral blood smear test revealed no changes in white blood cell morphology, a decreased neutrophil and lymphocyte count, a low red blood cell count with a high mean corpuscular volume, and anisocytosis. High ferritin and cobalamin levels were present in the biochemistry panel, and the folic acid concentration was normal. Renal and hepatic function tests, electrolytes, and coagulation markers were in normal ranges. Bone marrow aspiration and a trephine biopsy were obtained; marrow hypocellularity was confirmed, and the patient was diagnosed with aplastic anemia. Autoimmune disease panel and cytogenetical testing revealed no abnormalities in the normal female karyotype.

During the period from June 2021 to September 2021, patient's complete blood counts were stable, and observation without therapy was initiated. In October 2021 blood test showed severe anemia of hemoglobin 5 g/dL and very severe neutropenia of segmented neutrophil count <0.5 × 10^9^/L. The patient was admitted to the hematology ward for multiple red blood cell mass transfusions. Granulocyte-colony stimulating factor (filgrastim 480 mcg/0.8 mL) was injected subcutaneously 2x a day for 5 days. During admission, platelet counts dropped to 40 10e^3^/mm^3^, and platelet mass transfusions were initiated. The patient started receiving immunosuppressive therapy with CsA 100 mg daily divided into 2 doses; however, the patient's hematological condition did not significantly improve, and multiple platelet and red blood cell transfusions were necessary until April 2022. During the patient's stay in the stem cell and tissue transplant ward, ATG therapy was considered, although the decision about alloHSCT treatment was made in April 2022 [2, 3].

The patient had a history of anorexia nervosa and amenorrhea diagnosed in 2015 at the age of 15, had a hospitalization in psychiatric diagnosis regard, and was in remission until August 2021, when relapse occurred. By the time of hospitalization for stem cell transplant, the patient was determined to be underweight, with a body weight of 27 kg and a BMI of 11.8 kg/m^2^.

Admission of the patient to the bone marrow transplantation unit at Vilnius University Hospital, Santaros Klinikos, Vilnius, Lithuania, was on the 25th of July 2022. Hematological findings at the time of admission: white blood cells, 1.41 *∗* 10 ^ 9/L; low neutrophil count, 0.31 *∗* 10 ^ 9/L; low HgB, 75 g/L; low red blood cells, 2.53 *∗* 10 ^ 12/l; anisocytosis; low Plt count, 56 *∗* 10 ^ 9/l. The molecular test for Cytomegalovirus was negative, and Epstein-Barr virus concentration was positive at 220 copies/ml.

Nutritionist and psychiatrist consultations were ordered during the patient's hospital stay, and recommendations and therapy were prescribed as follows: parenteral nutrition of protein/carbohydrates/lipids/glucose 500 ml/24 h; enteral Nutrison 500 ml feeding via nasogastric tube; mirtazapine 15 mg p/o qHS; and olanzapine 5 mg/d.

The transplant was postponed for 2 days due to high mortality risks reaching 30–40% for the patient being in critical condition: severely malnourished and underweight; however, conditioning therapy was started on the 28th of April, 2022, regarding the patient's positive physical activity status. Before conditioning therapy, a nasogastric tube for enteral nutrition and a central venous catheter were inserted. Parenteral nutrition was administered for 2 weeks during hospitalization [4]. Conditioning therapy consisted of the fludarabine/cyclophosphamide/alemtuzumab protocol with premedication [5]. The dosage of drugs was strictly controlled due to the patient's low BMI: alemtuzumab i/v 3 mg −8 day, 17 mg −7 day, 20 mg from −6th to −3rd day prior to transplant, fludarabine 32 mg i/v from −7 to −4 day, and cyclophosphamide 530 mg i/v −5 to −2 day (with mesna for prevention of hemorrhagic cystitis) [6, 7].

**Figure 1:**
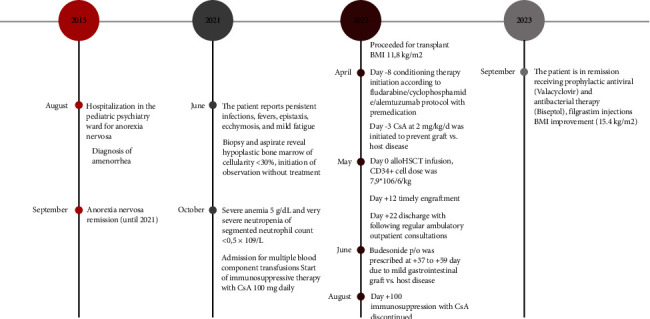
Timeline of diagnosis and treatment.

An alloHSCT with a donor-to-recipient HLA ratio of 10/10 was performed on the 6th of May 2022. Donor blood group B(II) RhD+, CMV+; recipient blood group AB RhD+, CMV+. The CD34+ cell dose was 7.9 *∗* 106/6/kg. No transfusion complications were experienced after the transplant. Timely engraftment was observed on +12 day with no clinically significant complications during the aplasia period, when white blood cell counts started to increase. CsA at 2 mg/kg/d was initiated to prevent GVHD at −3 day; the dose was increased titrated to 3-4 mg/kg/d to reach the target concentration of 200–400 ng/Ll. Budesonide p/o was prescribed at +37 to +59 day due to mild gastrointestinal GVHD which completely resolved. The CsA dose was slowly decreased and tapered during the posttransplant period from day +100 and stopped. Biseptol, valacyclovir, and ciprofloxacin were prescribed for infection prophylaxis during the posttransplant period. The patient was discharged on day +22 following regular ambulatory outpatient consultations for the period from June 2022 to February 2023 with an improvement of hematological status alongside weight gain of 7 kg (weight 34 kg, BMI 14.7 kg/m^2^). The patient's clinical history timeline is observed in Figure 1 (Figure 1 Timeline of diagnosis and treatment).

Currently, the patient is in remission and receiving prophylactic antiviral (Valacyclovir) and antibacterial therapy (Biseptol). HgB and Plt counts are normal, whereas G-CSF is occasionally needed due to episodes of neutropenia. Filgrastim 480 mcg/0.8 mL 3x weekly for neutrophil count improvement; no signs of infections; or GHVD are evident. Physically,clinically, and mentally the patient's status is positively improving.

## 3. Discussion

Nutrition is an important factor during alloHSCT treatment in malnourished patients, and the involvement of multiple healthcare professionals is a determining factor for a better outcome [4]. In multivariate analysis, subjective global assessment of severely malnourished patients receiving alloHSCT compared to well-nourished and moderately nourished patients remains an independent risk factor for acute GVHD and nonrelapse mortality, worse progression-free survival, worse overall 1-year survival, and worse overall survival [5, 8]. Our case represents that postalloHSCT development of acute GVHD in severely malnourished patients decreases and survival rates increase if proper multidisciplinary healthcare and cautious therapy monitoring are initiated.

Presumedly, no other literature reports describe a case of stem cell transplantation for severely underweight patients diagnosed with anorexia nervosa and aplastic anemia. Aplastic anemia is caused by bone marrow failure, resulting in low complete blood counts in peripheral smear and hypocellularity on marrow biopsy histological findings. Some literature findings mark out cases of malnourishment-caused cytopenias, whilst no reports have indicated severe aplastic anemia due to nutrition deficiency [9, 10]. Furthermore, the influence of malnutrition in patients receiving alloHSCT, conditioning regimen adaptation, and supportive therapy has not been described in previous literature findings.

Multiple literature sources have described the curative possibilities and low toxicity of hematopoietic stem cell transplantation for young patients diagnosed with severe aplastic anemia, therefore considering alloSCT the most effective standard of care [11].

Nevertheless, our patient was not considered a transplant candidate at the time of diagnosis due to her concomitant eating disorder and severe malnutrition. Only after the immunosuppressive therapy proved to be ineffective, which alongside genetic and autoimmune panel results confirmed the idiopathic etiology of aplastic anemia [12, 13], did the patient proceed to the matched unrelated donor alloSCT.

By the time of admission for alloSCT, the patient had lost more than 10% of body weight at diagnosis and presented to the unit with a BMI of 11,8 kg/m^2^, being critically underweight. The start of the conditioning was delayed for 2 days due to the necessity of multiple consults, although after careful assessment of the patient's clinical condition, physical abilities, and good compliance, the conditioning regimen was initiated. The conditioning regimen and medication doses were individualized on the patient's weight and clinical status to reduce the possibility of excessive toxicity [14]. Concomitant parenteral and enteral nutrition were administered with recommendations from dietologists and nutrition therapists [8, 15, 16]. Psychiatrist and psychologist consults were regular during the inpatient stay, and tetracyclic antidepressant (mirtazapine) and atypical antipsychotic (Olanzapine) were prescribed to reduce the psychiatric symptoms of anorexia nervosa and to avoid drug-drug interactions.

## 4. Conclusions

In conclusion, we report a case of successful alloHSCT for a patient with severe aplastic anemia and anorexia nervosa, who presented with a critically low BMI. Our case report highlights the importance of supportive measures of nutritional therapy and psychiatric treatment and emphasizes the multidisciplinary approach. This approach may improve alloSCT outcomes in patients with severe malnutrition and eating disorders.

## Data Availability

No new data were created or analyzed in this study. Data sharing does not apply to this article.
